# The acute effects of caffeine intake on time under tension and power generated during the bench press movement

**DOI:** 10.1186/s12970-019-0275-x

**Published:** 2019-02-18

**Authors:** Michal Wilk, Michal Krzysztofik, Adam Maszczyk, Jakub Chycki, Adam Zajac

**Affiliations:** 1 Department of Sports Training, Jerzy Kukuczka Academy of Physical Education, Katowice, Poland; 2 Department of Statistics and Methodology, Jerzy Kukuczka Academy of Physical Education in Katowice, Katowice, Poland

**Keywords:** Resistance exercise, Movement tempo, Eccentric contraction, Velocity, Repetition

## Abstract

**Background:**

The ability to generate high levels of power is one of the key factors determining success in many sport disciplines. Although there are studies confirming ergogenic effects of caffeine (CAF) on different physical and mental abilities, much controversy remains about its influence on power. The main goal of this study was to assess the effects of caffeine supplementation on time under tension (TUT) and the number of performed repetitions (REP). The second objective was to determine the effects of CAF supplementation on power (P) and movement velocity (V) during the bench press movement. Additionally the authors evaluated whether CAF has a significant effect on velocity of the bar in the eccentric (ECC) phase (VE_MEAN_) of the bench press movement.

**Methods:**

The study included 20 men (20–31 yrs., 87.3 ± 7.7 kg) with at least 2 years of experience in resistance training. The study participants were divided randomly into two groups: the supplemented group ingested caffeine before exercise (G_CAF_), while the control group was given a placebo (G_CON_). The exercise protocol consisted of performing the bench press movement with a load equal to 70%1RM with maximal possible velocity (X/0/X/0). The experimental sets were performed to momentary muscular failure.

**Results:**

The repeated measures ANOVA between the G_CAF_ and G_CON_ groups revealed statistically significant differences in 2 variables. Post-hoc tests demonstrated statistically significant differences in TUT when comparing the group supplemented with caffeine (13.689 s G_CAF_) to the one ingesting a placebo (15.332 s G_CON_) at *p* = 0.002. Significant differences were also observed in mean velocity during the eccentric phase of movement (0.690 m/s in the G_CAF_ to 0.609 in G_CON_ with p = 0.002). There were no significant differences in generated power and velocity in the CON phase of the movement between the G_CAF_ and G_CON._

**Conclusions:**

The main finding of the study is that CAF ingestion increases movement velocity of the bar in the eccentric phase of the movement, what results in shortening of the time under tension (TUT) needed for performing a specific number of repetitions, without decreasing power and velocity in the CON phase of the movement.

## Background

Resistance training and power are significant components of conditioning programs in competitive sports. The ability to generate high levels of power has been indicated as a determinant of success in sports that require an optimal ratio of strength to velocity when performing a motor activity [1, 2]. Optimization of resistance training and the pattern of adaptive changes related to the development of muscular strength and power have been the focus of interest of scientists from different fields of studies [3–6]. In addition to training, diet and supplementation also have a significant effect on adaptation and post-exercise responses [7–12]. To date, few supplements have been shown to have a direct ergogenic effect on physical capacity. Among them are caffeine, creatine monohydrate, sodium bicarbonate and beta alanine [13]. Although studies have confirmed the ergogenic effects of caffeine (CAF) in many aspects, much controversy remains about its effects on the power generated by the upper limbs [14, 15]. The most frequently consumed dose of caffeine during research with athletes ranges from 2 to 9 mg/kg body mass, ingested in the form of pills or capsules 30 to 90 min before exercise. Mechanisms responsible for ergogenic effects of caffeine are linked to the impact on various tissues, organs and systems, of the human body. In the central nervous system (CNS), CAF acts through interactions with adenosine receptors that influence the release of noradrenaline, dopamine, acetylcholine and serotonin [16–19] and, consequently, increase muscle tension [20]. Increased muscle activation can lead to a higher energy demand during exercise, thus leading to a faster depletion of energy substrates in muscle cells [21]. Caffeine can stimulate calcium release from the sarcoplasmic reticulum [22] and can also inhibit its reuptake [23]. Numerous studies have discussed the effects of caffeine intake on human physical fitness [24–30]. However, in regards to strength and power performance, the results of caffeine supplementation are ambiguous. Studies have shown faster neuromuscular conduction [31], increased motor unit activation [32, 33], and increased number of repetitions (REP) in the bench press following caffeine intake 60 min before exercise, compared with a placebo [33, 34]. Furthermore, Green et al. [35] failed to demonstrate significant differences in the number of REP between the placebo and CAF groups during the bench press and for the first and second set of the leg press exercise. No ergogenic effect of CAF on REP was also documented in a study by Grgic and Mikulic [36]. Considering the effects of CAF on the level of maximal strength, the results tend to be inconclusive. Goldstein et al. [18] demonstrated a significant effect of CAF supplementation on strength in a group of women. On the contrary, Astorino et al. [37], Beck et al. [15] did not find such an effect in a group of experienced strength athletes. One should emphasize that previous research on CAF supplementation and the level of strength and power, as well as the number of performed repetitions considered resistance exercises performed at volitional or maximal velocity (V) of the entire movement, without a precise control of movement tempo during the concentric (CON) and eccentric (ECC) phases. Movement tempo has been defined by seconds which correspond to individual movement cadences (ECC/pause/ CON/pause). Value X for the concentric movement represents maximal movement velocity. Changes in movement tempo during resistance training impacts exercise volume, the level of generated force, muscle power, and the rate of muscle hypertrophy [38–45]. The number of REP performed at a specific tempo impacts total time under tension (TUT) in a particular set. TUT provides accurate information about the duration of resistance effort for a set and for the entire training session. Wilk et al. [44] demonstrated significant differences in TUT and REP between the 2/0/2/0 and 5/0/3/0, as well as 6/0/4/0 tempos despite using the same external load and exercise to momentary muscular failure. That study demonstrated, that a greater number of performed REP was not synonymous with longer TUT. Therefore, TUT is an indicator of the work performed by the muscles in both, the ECC and CON phases of the movement. TUT determines how long the resistance effort lasts regardless of the number of REP performed. Previous studies regarding the ergogenic effects of caffeine on exercise volume, number of performed repetitions or the level of generated power did not consider exercise cadence, nor particular movement phases and TUT. This may explain the divergent results of studies that have used caffeine intake before resistance exercise. Furthermore, studies have failed to analyse the effect of CAF during controlled or variable movement tempo or cadence and the effect of these factors on strength, muscle power and training volume. It has not been demonstrated whether the ergogenic effect of caffeine concerns the entire movement or particular phases of the movement (CON and ECC).

Therefore, the main objective of this study was to assess the effects of caffeine intake on time under tension, the number of performed repetitions, and to determine the influence of CAF on muscular power (P) and movement velocity (V) in the CON phase. An additional goal of the study was to demonstrate the effects of CAF supplementation on mean velocity in the ECC (VE_MEAN_) phase of the bench press movement.

## Methods

All testing was performed in the Strength and Power Laboratory at the Jerzy Kukuczka Academy of Physical Education in Katowice. The experiment was performed following a randomized crossover, placebo-controlled, double-blind design, where each participant performed a familiarization session with a 1RM test on one day, and two different experimental sessions a week apart. The study participants were divided randomly into two groups: the group that ingested caffeine (G_CAF_) and the placebo group (G_CON_). Sixty minutes before each exercise protocol, CAF (5 mg/kg b.m.) or a placebo (all-purpose flour) were administered orally. CAF was provided in the form of standard capsules containing 300 mg of CAF, as well as those specifically prepared for the research, containing 50 and 5 mg doses of CAF. The placebo was provided in identical capsules as CAF. All CAF and placebo capsules were manufactured by Olimp Labs. Prior to the start of the study, an interview was conducted regarding caffeine intolerance, or any other physiological reactions to cafe intake. None of the subjects reported side effects in response to the use of CAF. The participants were instructed to follow their general nutrition and exercise protocols. All subjects completed questionnaires regarding health history, habitual caffeine intake from coffee, tea, soft drinks, chocolate, sport drinks, and caffeine-containing OTC drugs one week prior to testing. They were selected on the basis of their habitual caffeine consumption (< 200 mg/week) as determined by the questionnaire. In addition, subjects were asked to refrain from heavy exercise and alcohol consumption for 48 h before testing and to abstain from consuming caffeine-containing foods and beverages for 7 days prior to, and throughout, the study.

### Study participants

Twenty healthy strength trained men volunteered for the study after completing an ethical consent form (age = 25.7 ± 2.2 years, body mass = 87.3 ± 7.7 kg, bench press 1RM = 102,3 ± 8.5 kg; data presented as mean ± standard deviation [SD]) with a least one year of resistance training experience (2.3 ± 0.63 years; mean ± standard deviation [SD]). All study participants were over 18 years old. The participants were allowed to withdraw from the experiment at any moment, and were free of any pathologies or injuries. The study protocol was approved by the Bioethics Committee for Scientific Research, at the Academy of Physical Education in Katowice, Poland according to the ethical standards of the Declaration of Helsinki, 1983.

### Procedures

#### Familiarization session and one repetition maximum test

A familiarization session preceded the one repetition maximum testing. These two objectives were separated by a 15 min recovery period. The participants arrived at the laboratory at the same time of day as the upcoming experimental sessions (in the morning between 09:00 and 11:00 am) and cycled on an ergometer for 5 min at an intensity that resulted in a heart rate of around 130 bpm, followed by a general upper body warm-up. Next, the participants performed 15, 10, and 5 repetitions of the bench press exercise using 20, 40, and 60% of their estimated 1RM with a 2/0/X/0 cadence. The participants then executed single repetitions using a volitional cadence with a 5 min rest interval between successful trials. The load for each subsequent attempt was increased by 2.5 kg, and the process was repeated until failure. Hand placement on the barbell was individually selected with a grip width on the barbell of 150% individual biacromial distance (BAD) [46, 47]. BAD was determined by palpating and marking the acromion with a marker, and then measuring the distance between these points with a standard anthropometric tape [48]. The positioning of the hands was recorded to ensure consistent hand placement during all testing sessions. No bench press suits, weightlifting belts, or other supportive garments were permitted.

#### Experimental sessions

Two testing sessions were used for the experimental trials. The grip width was marked on the barbell with thin strips of athletic tape at the start of each session. The general and specific warm-up for the experimental sessions was identical to the one used for the familiarization session. After the warm up, participants started the main examinations and performed one set of the bench press to momentary failure with a load of 70%1RM. The eccentric and concentric phases were performed at maximal possible velocity (X/0/X/0). All repetitions were performed without bouncing the barbell off the chest, without intentionally pausing at the transition between the eccentric and concentric phases, and without raising the lower back off the bench. The interval between two stages of the experiment was 7 days. During the experimental trials the participants were encouraged to perform at maximal engagement according to the recommendations by Brown and Weir [49]. A linear position transducer system “Tendo Power Analyzer” (Tendo Sport Machines, Trencin, Slovakia) was used for the evaluation of bar velocity. The Tendo Power Analyzer is a reliable system for measuring movement velocity and power [50–56]. The system consists of a velocity sensor connected to the load by a kevlar cable which, through an interface, instantly transmits the vertical velocity of the bar to a specific software installed in the computer (Tendo Power Analyzer Software 5.0). The measurement was made independently in each repetition and automatically converted into the values of power (max, mean), concentric velocity (max, mean) and eccentric velocity (mean). All familiarization and experimental sessions were recorded by means of a Sony camera (Sony FDR191 AX53). Time under tension was obtained manually from the recorded data using slow speed playback (1/5 speed). In order to ensure the reliability of manual data collection, four independent persons made the data analysis from the Sony camera. There were no significant differences in the TUT [s] between the data collected by 4 evaluators. All participants completed the described testing protocol. The procedures were performed in identical environmental conditions, with an air temperature of 19.2 °C and humidity of 58% (Carl Roth hydrometer, Germany).

The following variables were registered:

- TUT - time under tension [s]
- REP - number of repetitions [n],
- P_MAX_ - maximal concentric power [W]
- P_MEAN_ - mean concentric power [W]
- V_MAX_ - maximal concentric velocity [m/s]
- V_MEAN_ - mean concentric velocity [m/s]
- VE_MEAN_ - mean eccentric velocity [m/s]

### Statistical analysis

The Shapiro-Wilk, Levene and Mauchly’s tests were used in order to verify the normality, homogeneity and sphericity of the sample data variances. Verification of differences between the G_CAF_ and G_CON_ after caffeine and placebo supplementation was performed using ANOVA with repeated measures. Effect sizes (Cohen’s d) were reported where appropriate. Parametric effect sizes were defined as large for d > 0.8, as moderate between 0.8 and 0.5, and as small for < 0.5 [57–59]. The statistical significance was set at *p* < 0.05. All statistical analyses were performed using Statistica 9.1 and Microsoft Office, and were presented as means with standard deviations.

## Results

The repeated measures ANOVA between the G_CAF_ and G_CON_ after caffeine and placebo supplementation revealed statistically significant differences between groups in 2 variables (Tables 1, 2).

**Table 1:** Results of the 7 considered variables in the group that ingested caffeine (G_CAF_) and the placebo group (G_CON_)

Variables	Caffeine group	Placebo group
Maximal concentric power [W]	750,8 ± 113,495	684,5 ± 110,12
Maximal concentric velocity [m/s]	0,81 ± 0,073	0,767 ± 0,071
Mean concentric power [W]	383 ± 51,9	354 ± 51,2
Mean concentric velocity [m/s]	0,467 ± 0,043	0,44 ± 0,0432
Number of repetitions [n]	16,6 ± 1,56	15,9 ± 1,44
Time under tension [s]	13,68 ± 1346 *	15,33 ± 1698 *
Mean eccentric velocity [m/s]	0,69 ± 0,088 *	0,609 ± 0,057 *

Notes: mean ± standard deviation [SD]; * statistically significant differences

**Table 2:** Differences in the 7 considered variables between the group that ingested caffeine (G_CAF_) and the placebo group (G_CON_)

Variables	D	F	P
Maximal concentric power [W]	0.245	3.52	0.068
Maximal concentric velocity [m/s]	0.231	3.48	0.070
Mean concentric power [W]	0.210	3.04	0.089
Mean concentric velocity [m/s]	0.221	3.60	0.065
Number of repetitions [n]	0.132	2.15	0.151
Time under tension [s]	0.903	11.49	0.002
Mean eccentric velocity [m/s]	0.912	11.64	0.002

Notes: d - effect size; p - statistical significance; F – value of analysis of variance function

An appropriate formula for comparing means in two groups of equal size is presented below:

$$ n=\frac{2}{d^2}x{c}_{p. power} $$

where *n* is the number of subjects required for each group, *d* is the standardized difference and *c*_*p*,*power*_ is a constant defined by the values chosen for the *P* value and power. The number of participants required in each trial to detect a standardized difference of 0.87 with 80% power using a cutoff for statistical significance of 0.05 is as follows:

$$ n=\frac{2}{0,{87}^2}x{c}_{\mathrm{0,05,80}\%}=\frac{2}{\mathrm{0,756}}x7,9=2,39x7,9=20,89 $$

Post-hoc tests revealed statistically significant differences for TUT [s], when comparing the CAF group (13.689 s) to the control group (15.332 s), at *p* = 0.002. Similar, statistically significant differences were observed for eccentric velocity: from 0.690 m/s in the G_CAF_ to 0.609 in G_CON_ at p = 0.002.

The average 1RM bench press results equaled 102,3 ± 8.5 kg.

## Discussion

The main finding of the study was that CAF supplementation has a significant effect on TUT [s], with no significant changes in the number of performed REP [n]. The ingestion of CAF led to a significant decrease in TUT during the explosive bench press exercise between the CAF group (G_CAF_) and the placebo group (G_PLA_). To date, no scientific study has analyzed the effect of caffeine on TUT. Despite significant differences in TUT between G_PLA_ and G_CAF_, it was not related to the number of performed REP. The evaluations showed no statistical significance in REP (G_CAF_ 16.6 ± 1.56 vs G_PLA_ 15.9 ± 1.69). This is inconsistent with previous findings [33, 35, 60, 61]. Duncan et al. [34] demonstrated an increase in exercise volume using an external load of 60%1RM, following the use of 5 mg of CAF/kg/b.m. In the next years, Duncan et al. [33, 60] also confirmed a significant effect of CAF intake on the number of performed REP, exercise volume and increase in muscle activity. The results of our study are partially consistent with those published by Goldstein et al. [18], who failed to find the effect of CAF supplementation on the number of performed REP. A similar pattern was observed in a study by Williams et al. [14], Green et al. [35], Astorino et al. [37], Richardson and Clarke [62]. However, one should stress that most studies that have analyzed the effect of CAF intake on exercise volume, both those that have demonstrated a significant effect on exercise capacity [33, 34, 37, 60, 63] and those which did not confirm such effects [14, 62], evaluated exercise volume using the number of REP or tonnage. No previous studies have taken into account movement tempo and TUT, during resistance exercises. According to Wilk et al. [44] TUT is a more accurate and credible indicator of work performed compared to the number of performed REP. These findings were also confirmed in our study, which showed a significant decline in TUT between the G_CAF_ and G_CON_ groups (13,68 ± 1346 vs 15,33 ± 1698 s) with no significant changes in the number of REP. The TUT determines how long the resistance effort lasts regardless of the number of REP performed. A decreased values of TUT in the CAF group may have resulted from the increased muscle tension generated during the movement in the CAF group. CAF leads to higher activation of motor units [33] and higher MVIC [20, 32]. Increased muscle activation can lead to a higher energy demand during exercise, thus leading to a faster depletion of energy substrates in muscle cells [21], which may partially explain a decline in TUT in the G_CAF_ group. However, the effect of increased muscle tension following CAF intake did not modify the power generated during the CON phase of the movement. The experiment did not demonstrate significant changes in P_MEAN_, P_MAX_ and V_MEAN_, V_MAX_, despite a significant shortening of TUT in the G_CAF_ group. The lack of significant differences in P and V indicates that the decline in TUT in the G_CAF_ group is not related to the level of generated power and velocity in the CON phase of the movement. Importantly, the experiment showed a significant increase in velocity but only in the ECC phase of the movement (VE_MEAN_). A significant increase in ECC velocity (G_CAF_ 0.690 ± 0.08 ms vs G_PLA_ 0.609 ± 0.05 ms) with a presumable increase in muscle tension in G_CAF_ can partially explain the decline in TUT. Cole et al. [64] and other authors suggested that caffeine impacts mechanoreceptors feedback, such as the Golgi apparatus and class III/IV muscle afferents [64–66]. Caffeine may alter feedforward information and may alter how information from either feedforward/feedbackward mechanism is processed centrally [67]. Despite the increase in velocity of the ECC movement, it did not lead to significant changes in velocity and power generated during the CON phase of the movement. Cronin et al. [68] demonstrated that muscle power generated during the CON movement depends on the effective use of the stretch-shortening cycle (SSC). The SSC cycle leads to the release of the energy stored in the ECC phase in order to maximize power generated during the CON phase. The same author [68] demonstrated that performing an ECC contraction before the CON phase impacts the generated power compared to performing only the concentric phase of the movement. A faster tempo of the entire movement cycle without a pause between the ECC and CON phases increases activation and more effective utilization of the SSC cycle [69]. However, to date, it remains unclear whether the efficiency of SSC depends on the velocity in the ECC phase and whether the SSC is susceptible to CAF supplementation. Although in this study VE_MEAN_ was higher in G_CAF_ (0.690 ± 0.08 ms vs 0.609 ± 0.05 ms), this change did not lead to a more effective use of the energy accumulated for explosive performance of the CON phase. Therefore, it can be speculated that the change in VE_MEAN_ and CAF intake do not lead to a more effective use of the SSC cycle. No increase in both mean (V_MEAN_; P_MEAN_) and maximal (V_MAX_; P_MAX_) values of CON movement with declining TUT questions the validity of CAF intake before high-intensity anaerobic exercise with a duration of between ten and twenty seconds. A limitation of the present study pertains to the lack of assessments related to CAF intolerance in the tested athletes. However it should be noted that before and during the experiment no study participant reported any side effects from ingesting caffeine.

## Conclusion

The results of the present study indicate that the use of CAF before exercise does not have a significant effect on the generated power and velocity of the CON phase of the movement. CAF ingestion increases movement velocity of the bar in the eccentric phase of the movement, what results in shortening of the time needed for performing a specific number of repetitions, without decreasing power and velocity of the CON phase of the movement. Our findings indicate that the value of TUT may be a more accurate and credible indicator of work performed during a resistance training session, compared to the number of performed repetitions and, consequently, may be a new variable used in the analysis of ergogenic aids on exercise capacity in athletes.

## Abbreviations

CAF: Caffeine
CON: Concentric
ECC: Eccentric
G_CAF_: Caffeine group
G_CON_: Placebo group
P: Power
REP: Repetition
SSC: Stretch-shortening cycle
TUT: Time under tension
V: Velocity
VE: Eccentric velocity

## References

[1] Baker D, Newton RU (2005). Acute effect on power output of alternating an agonist and antagonist muscle exercise during complex training. J Strength Cond Res..

[2] Argus CK, Gill ND, Keogh JWL, Hopkins WG (2014). Assessing the variation in the load that produces maximal upper-body power. J Strength Cond Res.

[3] Clarkson PM, Nosaka K, Braun B (1992). Muscle function after exercise-induced muscle damage and rapid adaptation. Med Sci Sports Exerc.

[4] Cramer JT, Housh TJ, Weir JP, Johnson GO, Ebersole KT, Perry SR (2002). Power output, mechanomyographic, and electromyographic responses to maximal, concentric, isokinetic muscle actions in men and women. J Strength Cond Res..

[5] Aaberg E (2006). Muscle mechanics.

[6] Ratamess NA, Alvar BA, Evetoch TK, Housh TJ, Kibler WB, Kraemer WJ (2009). Progression models in resistance training for healthy adults. Med Sci Sports Exerc.

[7] Wilk M, Michalczyk M, Golas A, Krzysztofik M, Maszczyk A, Zajac A (2018). Endocrine responses following exhaustive strength exercise with and without the use of protein and protein carbohydrate supplements. Biol Sport.

[8] Williams AG, Ismail AN, Sharma A, Jones DA (2002). Effects of resistance exercise volume and nutritional supplementation on anabolic and catabolic hormones. Eur J Appl Physiol.

[9] Bosse JD, Dixon BM (2012). Dietary protein to maximize resistance training: a review and examination of protein spread and change theories. J Int Soc Sports Nutr..

[10] Cermak NM, Res PT, de Groot LCPGM, Saris WHM, van Loon LJC (2012). Protein supplementation augments the adaptive response of skeletal muscle to resistance-type exercise training: a meta-analysis. Am J Clin Nutr.

[11] Burke LM (2017). Practical issues in evidence-based use of performance supplements: supplement interactions, repeated use and individual responses. Sports Med.

[12] Maughan RJ, Burke LM, Dvorak J, Larson-Meyer DE, Peeling P, Phillips SM (2018). IOC consensus statement: dietary supplements and the high-performance athlete. Br J Sports Med.

[13] Kerksick CM, Wilborn CD, Roberts MD, Smith-Ryan A, Kleiner SM, Jäger R (2018). ISSN exercise & sports nutrition review update: research & recommendations. J Int Soc Sports Nutr..

[14] Williams A, Cribb P, Cooke M, Hayes A (2008). The effect of ephedra and caffeine on maximal strength and power in resistance-trained athletes. J Strength Cond Res..

[15] Beck TW, Housh TJ, Schmidt RJ, Johnson GO, Housh DJ, Coburn JW (2006). The acute effects of a caffeine-containing supplement on strength, muscular endurance, and anaerobic capabilities. J Strength Cond Res..

[16] Daly JW, Shi D, Nikodijevic O, Jacobson KA (1994). The role of adenosine receptors in the central action of caffeine. Pharmacopsychoecologia.

[17] Davis JM, Zhao Z, Stock HS, Mehl KA, Buggy J, Hand GA (2003). Central nervous system effects of caffeine and adenosine on fatigue. Am J Physiol Regul Integr Comp Physiol.

[18] Goldstein E, Jacobs PL, Whitehurst M, Penhollow T, Antonio J (2010). Caffeine enhances upper body strength in resistance-trained women. J Int Soc Sports Nutr.

[19] Ferré S (2016). Mechanisms of the psychostimulant effects of caffeine: implications for substance use disorders. Psychopharmacology.

[20] Behrens M, Mau-Moeller A, Weippert M, Fuhrmann J, Wegner K, Skripitz R (2015). Caffeine-induced increase in voluntary activation and strength of the quadriceps muscle during isometric, concentric and eccentric contractions. Sci Rep.

[21] Bogdanis GC (2012). Effects of physical activity and inactivity on muscle fatigue. Front Physiol.

[22] Supinski GS, Deal EC, Kelsen SG (1984). The effects of caffeine and theophylline on diaphragm contractility. Am Rev Respir Dis.

[23] Endo M (1977). Calcium release from the sarcoplasmic reticulum. Physiol Rev.

[24] Burke LM (2008). Caffeine and sports performance. Appl Physiol Nutr Metab.

[25] Davis JK, Green JM (2009). Caffeine and anaerobic performance: ergogenic value and mechanisms of action. Sports Med.

[26] Graham TE (2001). Caffeine and exercise: metabolism, endurance and performance. Sports Med.

[27] Magkos F, Kavouras SA (2005). Caffeine use in sports, pharmacokinetics in man, and cellular mechanisms of action. Crit Rev Food Sci Nutr.

[28] Rossi FE, Panissa VLG, Monteiro PA, Gerosa-Neto J, Caperuto ÉC, Cholewa JM (2017). Caffeine supplementation affects the immunometabolic response to concurrent training. J Exerc Rehabil.

[29] Tarnopolsky MA (2010). Caffeine and creatine use in sport. Ann Nutr Metab.

[30] Warren GL, Park ND, Maresca RD, McKibans KI, Millard-Stafford ML (2010). Effect of caffeine ingestion on muscular strength and endurance: a meta-analysis. Med Sci Sports Exerc.

[31] Bazzucchi I, Felici F, Montini M, Figura F, Sacchetti M (2011). Caffeine improves neuromuscular function during maximal dynamic exercise. Muscle Nerve.

[32] Park ND, Maresca RD, McKibans KI, Morgan DR, Allen TS, Warren GL (2008). Caffeine enhancement of maximal voluntary strength and activation in uninjured but not injured muscle. Int J Sport Nutr Exerc Metab.

[33] Duncan MJ, Thake CD, Downs PJ (2014). Effect of caffeine ingestion on torque and muscle activity during resistance exercise in men. Muscle Nerve.

[34] Duncan MJ, Oxford SW (2011). The effect of caffeine ingestion on mood state and bench press performance to failure. J Strength Cond Res..

[35] Green JM, Wickwire PJ, McLester JR, Gendle S, Hudson G, Pritchett RC (2007). Effects of caffeine on repetitions to failure and ratings of perceived exertion during resistance training. Int J Sports Physiol Perform..

[36] Grgic J, Mikulic P (2017). Caffeine ingestion acutely enhances muscular strength and power but not muscular endurance in resistance-trained men. Eur J Sport Sci..

[37] Astorino TA, Rohmann RL, Firth K (2008). Effect of caffeine ingestion on one-repetition maximum muscular strength. Eur J Appl Physiol.

[38] Hatfield DL, Kraemer WJ, Spiering BA, Häkkinen K, Volek JS, Shimano T (2006). The impact of velocity of movement on performance factors in resistance exercise. J Strength Cond Res..

[39] Headley SA, Henry K, Nindl BC, Thompson BA, Kraemer WJ, Jones MT (2011). Effects of lifting tempo on one repetition maximum and hormonal responses to a bench press protocol. J Strength Cond Res..

[40] Hunter GR, Seelhorst D, Snyder S (2003). Comparison of metabolic and heart rate responses to super slow vs. traditional resistance training. J Strength Cond Res..

[41] Keeler LK, Finkelstein LH, Miller W, Fernhall B (2001). Early-phase adaptations of traditional-speed vs. superslow resistance training on strength and aerobic capacity in sedentary individuals. J Strength Cond Res..

[42] Sakamoto A, Sinclair PJ (2006). Effect of movement velocity on the relationship between training load and the number of repetitions of bench press. J Strength Cond Res.

[43] Westcott WL, Winett RA, Anderson ES, Wojcik JR, Loud RL, Cleggett E (2001). Effects of regular and slow speed resistance training on muscle strength. J Sports Med Phys Fitness.

[44] Wilk M, Golas A, Stastny P, Nawrocka M, Krzysztofik M, Zajac A (2018). Does tempo of resistance exercise impact training volume?. J Hum Kinet.

[45] Wilk M, Stastny P, Golas A, Nawrocka M, Jelen K, Zajac A (2018). Physiological responses to different neuromuscular movement task during eccentric bench press. Neuro Endocrinol Lett.

[46] Wagner LL, Evans SA, Weir JP, Housh TJ, Johnson GO (1992). The effect of grip width on bench press performance. Int J Sport Biomech.

[47] Green CM, Comfort P (2007). The affect of grip width on bench press performance and risk of injury. J Strength Cond Res..

[48] Clemons JM, Aaron C (1997). Effect of grip width on the myoelectric activity of the prime movers in the bench press. J Strength Cond Res.

[49] Brown LE, Weir JP (2001). ASEP procedures recommendation I: accurate assessment of muscular strength and power. J Exerc Physiol Online.

[50] Cormie P, McBride JM, McCaulley GO (2007). Validation of power measurement techniques in dynamic lower body resistance exercises. J Appl Biomech.

[51] Garnacho-Castaño M, López-Lastra S, Maté-Muñoz J (2015). Reliability and validity assessment of a linear position transducer. J Sports Sci Med.

[52] Goldsmith JA, Trepeck C, Halle JL, Mendez KM, Klemp A, Cooke DM (2018). Validity of the open barbell and tendo weightlifting analyzer systems versus the optotrak certus 3D motion capture system for barbell velocity. Int J Sports Physiol Perform.

[53] Gray M, Paulson S (2014). Developing a measure of muscular power during a functional task for older adults. BMC Geriatr.

[54] Jennings CL, Viljoen W, Durandt J, Lambert MI (2005). The reliability of the FitroDyne as a measure of muscle power. J Strength Cond Res..

[55] Jones RM, Fry AC, Weiss LW, Kinzey SJ, Moore CA (2008). Kinetic comparison of free weight and machine power cleans. J Strength Cond Res..

[56] Stock MS, Beck TW, DeFreitas JM, Dillon MA (2011). Test-retest reliability of barbell velocity during the free-weight bench-press exercise. J Strength Cond Res..

[57] Cohen J (1988). Statistical power analysis for the behavioral sciences.

[58] Maszczyk A, Golas A, Pietraszewski P, Roczniok R, Zajac A, Stanula A (2014). Application of neural and regression models in sports results prediction. Procedia Soc Behav Sci.

[59] Maszczyk A, Golas A, Czuba M, Krol H, Wilk M, Stastny P, Goodwin J, Kostrzewa M, Zajac A (2016). EMG analysis and modelling of flat bench press using artificial neural networks. S Afr J Res Sport Ph.

[60] Duncan MJ, Stanley M, Parkhouse N, Cook K, Smith M (2013). Acute caffeine ingestion enhances strength performance and reduces perceived exertion and muscle pain perception during resistance exercise. Eur J Sport Sci.

[61] Arazi H, Hoseinihaji M, Eghbali E (2016). The effects of different doses of caffeine on performance, rating of perceived exertion and pain perception in teenagers female karate athletes. Braz J Pharmaceut Sci.

[62] Richardson DL, Clarke ND (2016). Effect of coffee and caffeine ingestion on resistance exercise performance. J Strength Cond Res..

[63] Goldstein ER, Ziegenfuss T, Kalman D, Kreider R, Campbell B, Wilborn C (2010). International society of sports nutrition position stand: caffeine and performance. J Int Soc Sports Nutr.

[64] Cole KJ, Costill DL, Starling RD, Goodpaster BH, Trappe SW, Fink WJ (1996). Effect of caffeine ingestion on perception of effort and subsequent work production. Int J Sport Nutr.

[65] Jami L (1992). Golgi tendon organs in mammalian skeletal muscle: functional properties and central actions. Physiol Rev.

[66] Kaufman MP, Longhurst JC, Rybicki KJ, Wallach JH, Mitchell JH (1983). Effects of static muscular contraction on impulse activity of groups III and IV afferents in cats. J Appl Physiol Respir Environ Exerc Physiol.

[67] Plaskett CJ, Cafarelli E (2001). Caffeine increases endurance and attenuates force sensation during submaximal isometric contractions. J Appl Physiol.

[68] Cronin JB, McNair PJ, Marshall RN (2001). Magnitude and decay of stretch-induced enhancement of power output. Eur J Appl Physiol.

[69] Malisoux L, Francaux M, Nielens H, Theisen D (2006). Stretch-shortening cycle exercises: an effective training paradigm to enhance power output of human single muscle fibers. J Appl Physiol.

